# Experimental investigation on characterization of friction stir processed AZ31-based composite

**DOI:** 10.1038/s41598-024-66379-1

**Published:** 2024-07-04

**Authors:** Chaman Jeet Singh, Baljinder Ram, Jashanpreet Singh, Chander Prakash, Prabhu Paramasivam, Rahul Kumar

**Affiliations:** 1 Department of Mechanical Engineering, Punjabi University, Patiala, India; 2 University Center for Research and Development, Chandigarh University, Mohali, Punjab 140413 India; 3 Department of Research and Innovation, Saveetha School of Engineering, SIMATS, Chennai, Tamilnadu 602105 India; 4 Department of Mechanical Engineering, Mattu University, 318 Mettu, Ethiopia

**Keywords:** Friction stir processing, Metal joining, Mg alloys, AZ31 magnesium, Dissimilar joining, Alloys joining, Metal joining processes, Mechanical engineering, Composites

## Abstract

Present study has been conducted to characterize the Mg alloy namely AZ31-based composite joined by Friction stir processing (FSP) technique. This study deals with the effect of single and double passes in FSP of AZ31 Mg alloy. The single pass run in FSP is followed at tool rotation speed (N) of 1000 to 1400 rpm. Also, the double pass run in FSP was followed at these speeds without using reinforcements. The feedstock particles namely SiC, Al_2_O_3_, Cr, and Si powders were used in fabrication process. The hardness, impact strength, and tensile strength characteristics were assessed in the stir region zone, and the results indicated significant improvement in these properties. The highest values of mechanical strength were seen in the FSPed area with N = 1000 rpm at a constant transverse speed (*r*) of 40 mm/min. Also, the tensile strength of the two passes FSPed plates is much higher than that of the single section without any reinforcement, as revealed in previous study also. The Scanning electron microscopy (SEM) analysis is done at two different magnifications for the Silicon carbide, Alumina, Chromium, and Silicon powder reinforced composites fabricated at speed of 1000 rpm. The microstructure shows that reinforced particles were uniform dispersed into FSPed region and agglomerated with Mg matrix. Si powder produces finer microstructure as compare to SiC, Al_2_O_3_, Cr. FSP decreases the grain size of processed material. Optical Microscopy results revealed that the reinforcement particle produced a homogenous microstructure and, a refined grain and equally dispersed in matrix material without split to the particle.

## Introduction

Magnesium (Mg) is a common element in alloys made with a number of other metals. AZ31 is 1/3 lighter than aluminium, and 2/3 than steel^1,2^. The uniformity of applications of Mg alloys in a wide range of applications because of properties such as light weight, deformation properties, higher indentation, high impact resistance as well as fatigue resistance^3,4^. However, the AZ31 magnesium plate has extensive use across diverse sectors such as automobiles, aerospace, electronic devices, and medical^5–8^. Within the aerospace sector, it finds use in the production of helicopters and components for aeroplanes. Within the automobile sector, this technique is used for the manufacturing of engine components, gearboxes, and suspension elements. In the electronics sector, it is used for the production of portable gadgets like laptops and cellphones. Within the medical sector, this material is used for the fabrication of surgical equipment, plates, and implants.

In industry various types of forming^9,10^, casting/sintering^11^, advanced high precision welding^12,13^, and coatings^14^ are used in industry to manufacture various types of products. Advanced joining processes like advances welding^15^, wire-arc additive manufacturing^16,17^, friction stir processing^18^, and friction stir welding^19^ are widely used processed to joining and materials development of the dissimilar metals and alloys. However, the development of composites, their processing, and joining are quite tough processes^20–24^. In earlier years, Christener and Sylva^25^ had performed Friction stir welding on aluminium 2014AT-6 alloy and investigated mechanical properties on the effect of joint gap through parameter tolerance technique. Darras^26^ had performed an experimental as well as analytical study on aluminium 5052 alloy and investigated the microstructural and mechanical characteristics. They found that grain size structure, hardness is depends upon rotational and translational speeds and microstructure and properties depends upon temperature achieved during FSP. The microstructure of the materials highly relies on the transformational temperature during the process^27–31^. Morisada et al.^32^ performed a study to investigate the effect of reinforcement multi-walled carbon nanotubes (MWCNTs) dispersion on Friction stir processing with base metal matrix of aluminium AZ31 allloy under the constant tool rotating speed and three different travel rates. It was investigated that the high travel rates developed the sufficient flow of heat to yield a suitable viscosity in the AZ31matrix for the distribution of the MWCNTs. Chang et al*.*^33^ investigated the various characteristics of the Mg_35-70_ Al_5-25_ Zn_25-45_ multi-element intermetallic alloys fabricated by three or more passes of friction stir processing. They observed that the average hardness of the multi-element increased on vicker’s hardness scale and more refined and uniform microstructure obtained with increasing FSP passes. Dwivedi et al.^34^ adopted a FSP technique for the fabrication of Al-based composite utilizing a Spent Al_2_O_3_ catalyst (SAC) waste. They investigated that the developed Al/SAC composite showed fair distribution of constituent elements during FSP with superior tensile strength and hardness. Chen and Nakata^35^ performed friction stir welding on AZ31-alloy and Zn-coated steel to examine the microstructures and mechanical properties of the lap welded joints. The shear tensile strength results of lap joint show that welding speed significantly influences the failure loads of the joints at the high value of *N* and in terms of microstructure properties, the implementation of applying zinc coat result in development of Mg-Zn low-melting-point eutectic structure at the interfacial region. Kurt et al*.*^36^ performed an experimental study on friction stir processing on pure Al reinforced with SiC powder at various tool rotations and traverse rates. The result showed that the micro-hardness of composite surface improved thrice compared to that of pure Al as rotating and traverse rate increased and fine microscopic results observed. Antony et al.^37^ performed an experimental and analytical study on FSP of pure Al and AA6061 alloy plate and used an external filler plate. From grain size measurement and salt spray corrosion test, the results show the improved corrosion resistance by the coating of commercial Al plate. Wais et al*.*^38^ conducted a study to examine the effect of FSP on sand casting hypereutectic pure aluminium under the effect of parameters (such as* r* and *N*). Result showed as refined microstructure and much improved tensile and impact strength, and minor improvement in micro-hardness. Kumar et al.^39^ studied the role of FSP parameters on micro-hardness of Al/B4C composite. They observed that the tool's square pin profile, reduced inclination angle, and increased rotational speed resulted in a more uniform dispersion of B_4_C content with an increased microhardness. They also found that the Al-6063/B_4_C surfaces had 30% higher microhardness than the Al-6063 alloy. Devraju et al*.*^40^ tested of the effect of post-process artificial aging (PPAA) on tensile properties of FSPed SiC reinforced AA6061-T6 composite. Results were improved in terms of tensile properties and micro-hardness. Aonuma and Nakata^41^ experimental study on the Al alloys joints developed using Friction Stir Welding (FSW). They found that the the Ti/Al-2024 joint's tensile strength was found as 311 MPa which was greater than the Ti/Al-7075 FSW joint. Using a conical pin and a 50% overlap, Sinhmar et al*.*^42^ investigated the friction stir processing of Al-Zn-Mg alloy (AA-7039) plate. The surface that was produced was studied for its macrostructure, microstructure, tensile characteristics, and enhanced ductility and hardness. The AZ31 surface composite plate was manufactured by Huang et al*.*^43^ using the DFSP tool, which stands for direct friction stir processing. The morphological results indicated that the feedstock particles were uniformly disseminated in the agitation zone, and the micro-hardness improved while the grain size decreased as a consequence of the reinforcement. Kumar and Thansekhar^44^ conducted a study on the synergistic effect of FSW and FSP on two different Al alloys namely AA6101-T6 and AA1350. They discovered that narrower grooves showed the same distribution and mixing of Al_2_O_3_ in Al alloys. Kumar et al.^45^ examined the impact of Al_2_O_3_ and SiC on the stirred zone of dissimilar Al alloys during FSW. Results showed that the 100% SiC reinforcement led toward the poor mechanical and wear properties than the 100% Al_2_O_3_. Dwivedi et al.^46^ developed the eggshell reinforced composites utilizing the electromagnetic stir casting process. They found also reported the uniform distribution of eggshells. However, FSP provides rough surfaces which can be finished by using various types of processes like rolling, polishing, texturing, post-processing, etc.^47–51^.

Literature survey points towards the direction that the most of studies deals with the friction stir welding and there is lot of work which has necessary to carry out in Friction stir processing. The most of work has been processed on Aluminium by varying reinforcement in FSP but the number of reinforcements was restricted to one or two. So, present study deals with Magnisium composite fabricated by using four different reinforcements. The reinforcements used are Silicon carbide^40^, Alumina, Chromium and Silicon powder. Experimental procedure is performed on vertical milling machine^26^. Tool used for FSP is of cylindrical profile^18^. The FSP is performed at constant transverse speed and varying the rotational speed^32^. Investigation of mechanical properties including the hardness, impact strength and tensile strength is graphical representation. The microscopic images are obtained at two different magnifications for each specimen of specific reinforcement.

## Materials and methods

### Materials

**Figure 1:**
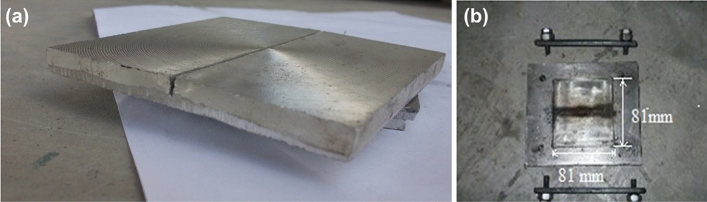
Groove cutting before starting the FSP process.

In fabrication process, the feedstock powders such as commercially available SiC, Al_2_O_3_, Cr and Si (99.9%) powder are used. For the purpose of the experiment, the matrix material that was used was AZ31 magnesium alloy. AZ31 magnesium plate (80 × 80 × 8 mm) in rolling state is used in this study. In order to produce these magnesium ingots, a power hacksaw was used to cut them into little pieces with nominal dimensions of 80 mm by 80 mm by 8 mm, as shown in Fig. 1a.

### Composite fabrication technique

**Figure 2:**
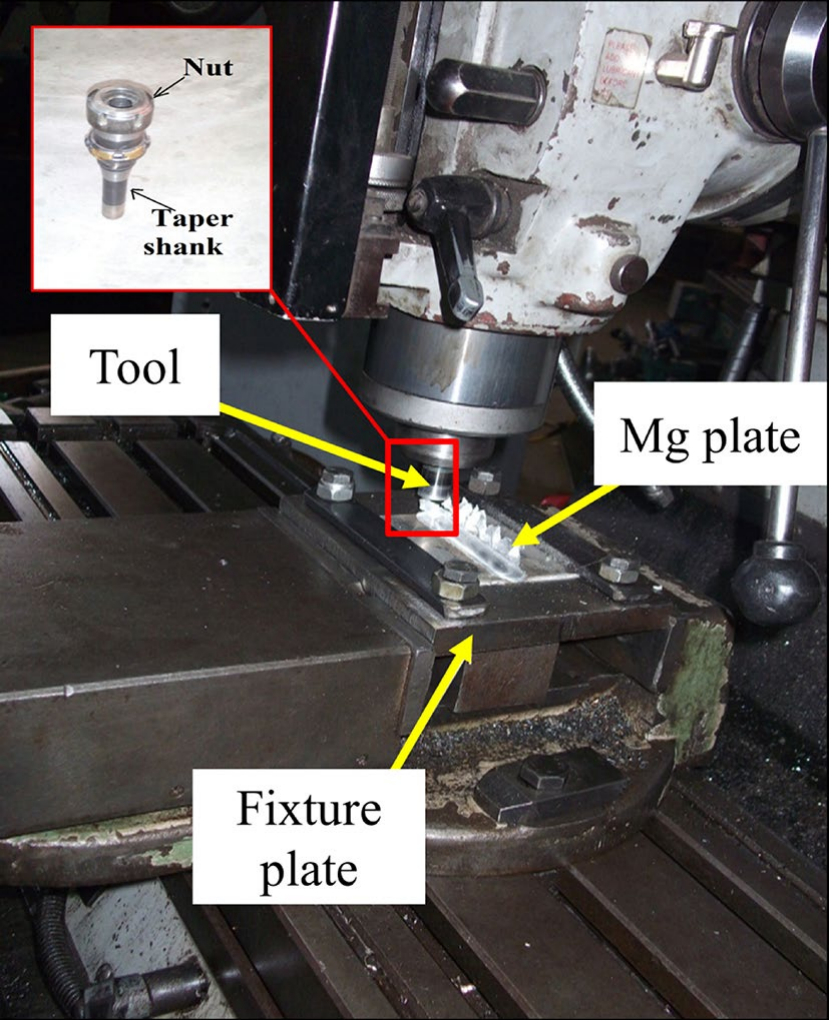
Tool profile and geometry.

The FSP technique in conjunction with a semi-automatic vertical milling machine is used to assemble the magnesium metal matrix composite. A brief overview of the fabrication process includes the tool design and placement, friction and heating, stirring and plastic deformation, reinforcement incorporation, and fabrication of surface composites^18,52,53^. The experimental setup of vertical milling machine is shown in Fig. 2. In this process, various parameters like tool design geometry, transverse speed of tool, tool rotation, and downward vertical force and the reinforcements are applied for purpose of significant results on the material flow sample and temperature circulation. These parameters thereby influence the microstructural development of material.

**Figure 3:**
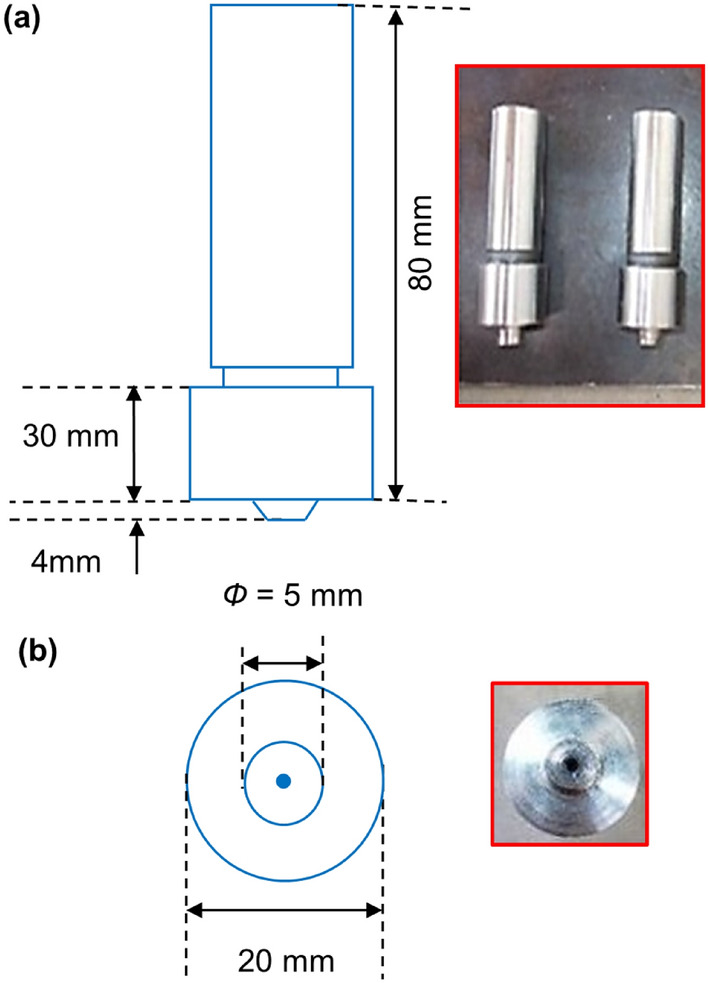
Tool profile and geometry.

Additionally, the tool arbour holding spindle and axis of the vertical milling machine are orientated in a vertical direction, as shown in Fig. 3. This machine is used for FSP of magnesium sample plates. The tool is also held in a vertical position, which is characteristic of the manual chuck. Milling cutters are rotated on the axis of the spindle while they are kept in place. In most cases, the spindle may be extended (or the table can be lifted or lowered, in order to achieve the same effect), which enables plunge cuts and drilling to be performed. Holding the tool in the required axis in the machine is the primary purpose of the tool holder, and the ER-Style Collets tool holder is the one that is employed. The fixture plate used for FSP work consists of rectangular base proportions 200 × 150 × 20 mm plate and two steel bars are bolted to a machined base plate by 2 bolts M15. The condition is given by 2 smaller bolts that press down an assembly of plates, yielding a uniformly distributed force along the work piece or matrix and the base plate is bolted to the machine worktable of the FSP equipment. The fixture system is of square dimension i.e. 81 × 81 mm, as shown in Fig. 1b. The specifications of FSP tool used are certain as below in Table 1. The tool used in this study has been illustrated in Fig. 3.

**Table 1:** Parameters of machine tool.

Tool parameters	Shoulder length	Shoulder diameter	Pin length	Pin diameter	Material	Hardness
Description	80 mm	20 mm	4 mm	5 mm	HSS	HRc 62

### Machining process

**Figure 4:**
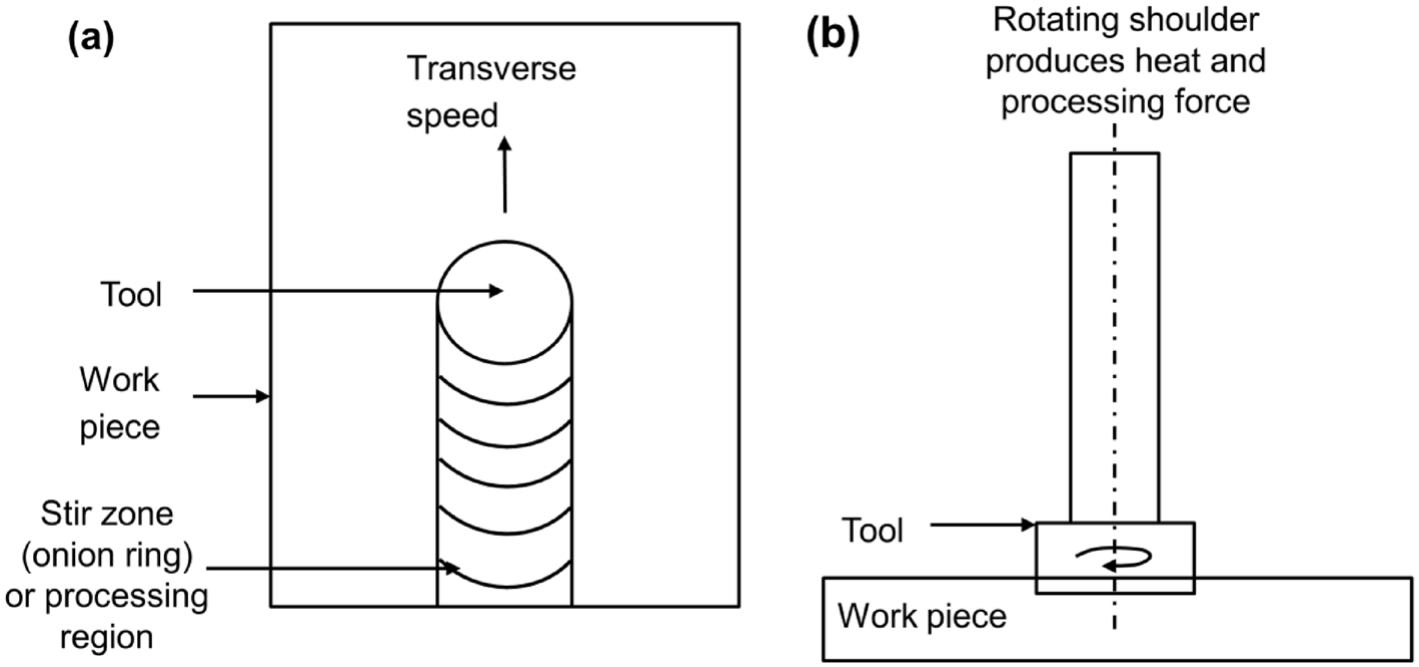
Schematic diagram of FSP showing (**a**) top view of transverse movement of tool, (**b**) front view of rotational movement of tool.

Before starting the machining process, a groove has been cut with 2 mm width and 5 mm depth on the Mg-matrix composite and channel or groove is placed at the edge of pin in the advancing side and center of the matrix plate as shown in Fig. 1a. To keep the various components from falling out of the groove, a cylindrical tool with a shoulder is used to seal it. The groove is then filled with SiC, Al_2_O_3_, Cr, and Si-powder particles. Kerosene oil is added to the particle powder so that it cannot be removed from the plate by air during operation and proper matrix mixing. With a single pass run, the following FSP settings are used: a constant tool travelling speed of 40 mm/min, a rotation speed of 1000 rpm, and a rotation speed of 1200 rpm. The double pass run in FSP was followed at these speeds without using feedstock. In this manner, a total of 12 plates undergo to FSP passes with different feedstocks and speed. Figure 4a and b illustrates the transverse movement of tool and the rotational movement of tool respectively.

### Materials characterization

**Figure 5:**
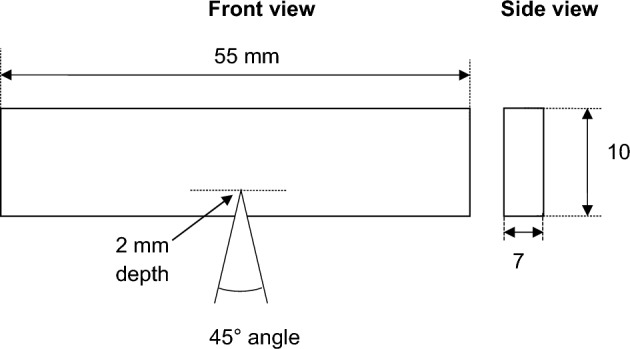
Model of specimen for impact test.

**Figure 6:**
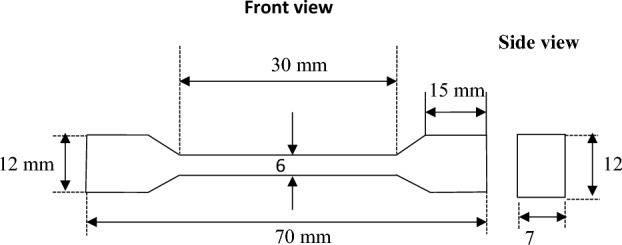
Model of specimen for tensile test.

Materials characterization includes the mechincal and microstructural analysis. This investigation used a Rockwell hardness machine to test the hardness of AZ31 and 12 FSPed plate samples. Each composite undergoes to 3 trails during hardness test and the mean of these values is taken for accuracy purpose. Impact strength is tested on Charpy impact testing machine. For Charpy test, the dimensions of specimen are 55 × 10 × 7 mm. The specimens are V- notched at angle 45˚ and depth of V is 2 mm. The specimen geometry for impact strength testing is shown in Fig. 5. For tensile test, the sample is fixed in the grips of universal machine in such a manner that the pull is applied axially. The cross section of specimen is rectangular shaped with dimensions of 70 × 12 × 7 mm as shown in Fig. 6.

The microscopic results contain Scanning electron microscopy and Optical microscopy based analysis. The Scanning electron microscopic analysis for the Al_2_O_3_, and Cr reinforced composites is performed at magnification of × 100 and × 1000, for SiC and Si-powder reinforced at magnification of × 100 and × 500 fabricated at speed of 1000 rpm. Optical Microscopy is conducted at magnification of × 500 for AZ31 and SiC reinforced composite fabricated at 1000 rpm.

## Results and discussion

The mechanical and microstructural properties have been investigated. Mechanical investigation includes Hardness, tensile strength and impact strength tests. Microstructural investigation includes the Scanning electron microscopy (SEM) and Optical microscopy (OM). The results from the analysis are described below in details.

### Visual analysis

**Figure 7:**
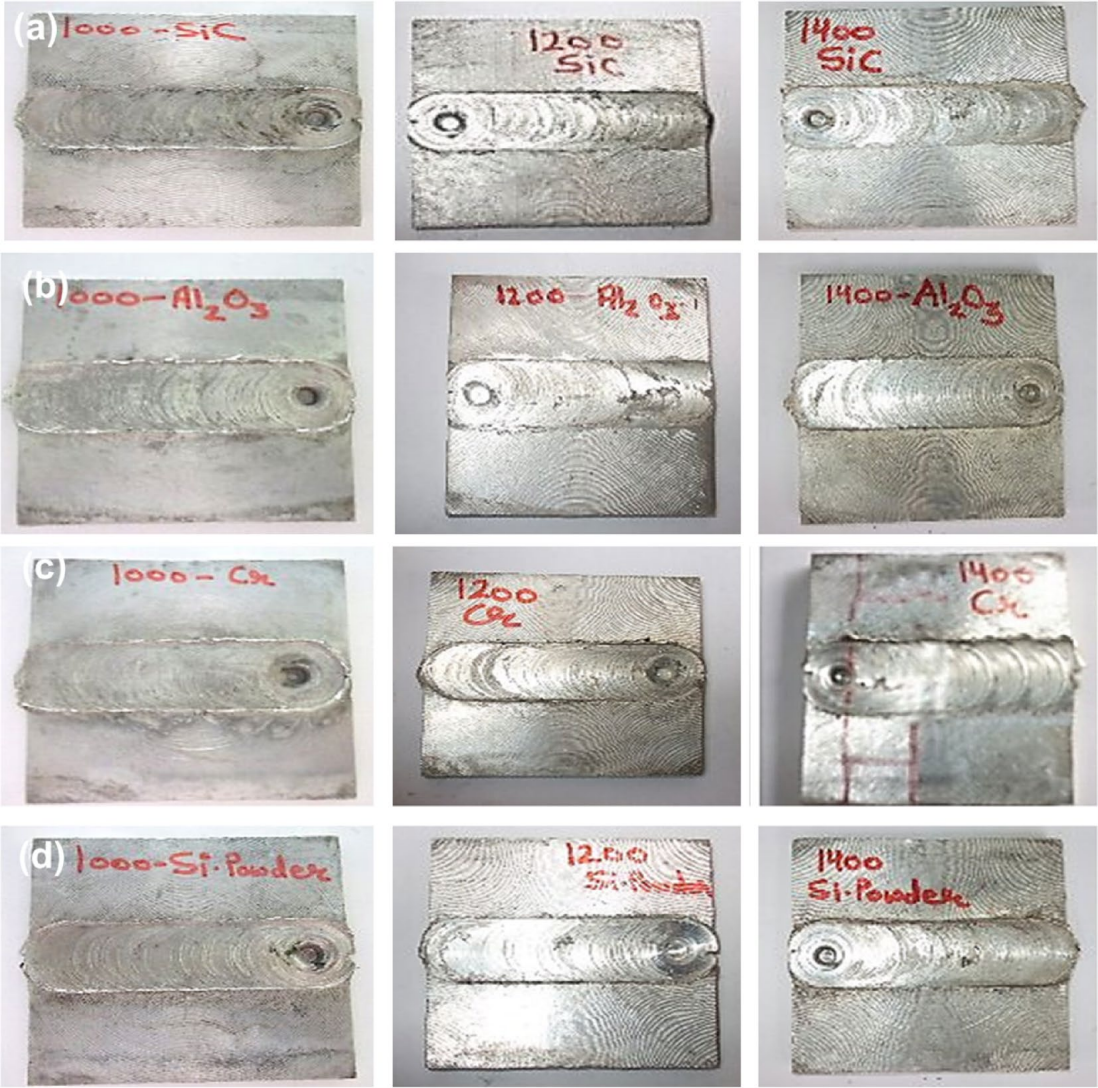
FSPed plates of (**a**) SiC (**b**) Al_2_O_3_ (**c**) Cr (**d**) Si-Powder; at 1000 rpm, 1200 rpm and 1400 rpm speeds after single pass run**.**

**Figure 8:**
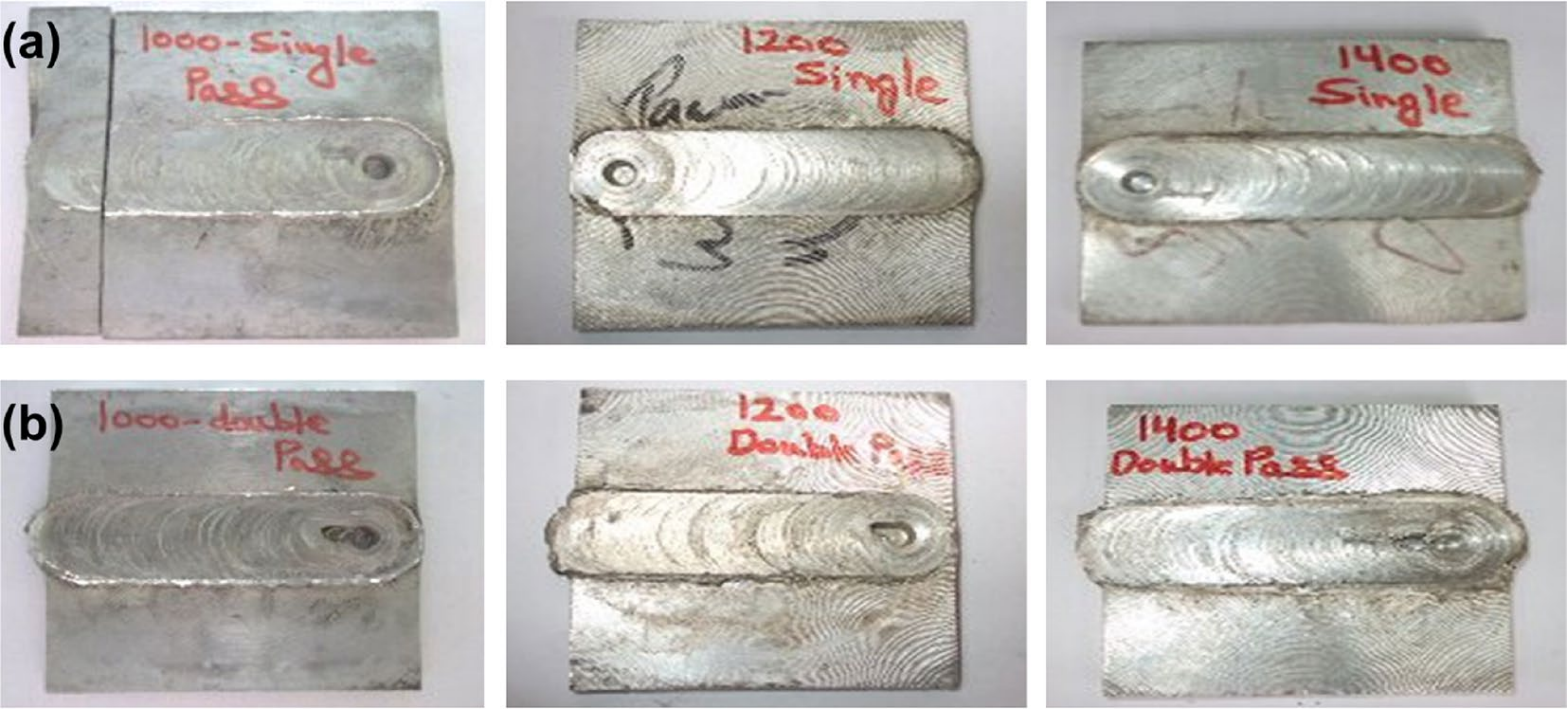
FSPed Mg plates of at 1000 rpm, 1200 rpm and 1400 rpm speeds after (**a**) single pass and (**b**) double pass run without using reinforcements, experiencing friction stir welding**.**

Sample specimens are made by single pass as well as double pass FSP. Figure 7 shows the FSP processed plates containing reinforced region of different reinforcements after single pass run. The base plate fabricated through single pass with reinforcements SiC, Al_2_O_3_, Cr and Silicon powder are shown in Fig. 7. It was perceived that the samples were embedded together and seems to be bonded well visually. Figure 8 shows the FSP processed plates without reinforcements after single and double pass run. It can be seen that the FSP plate was joined together smoothly without any imperfection here, the friction stir welding phenomenon comes into play.

### Hardness

**Figure 9:**
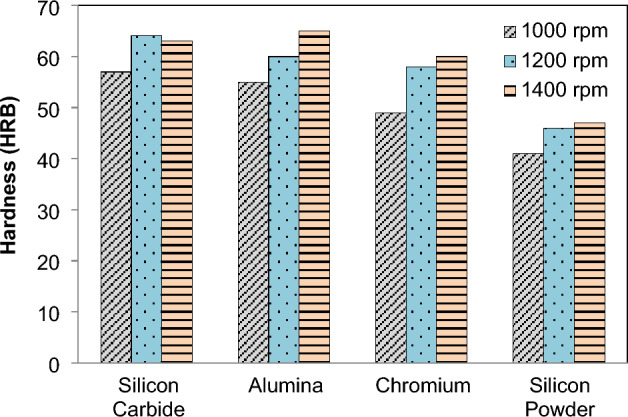
Effect of *N* on hardness.

The average hardness of magnesium alloy was determined to be 32 HRB in the hardness test. Because the treated plate has a fine grain microstructure, which increases hardness compared to unprocessed base plate18, FSPed reinforced sample plates exhibit superior hardness to base metal^18^. Figure 9 shows that the lower value of hardness in the stirred region at lowest Speed i.e. 1400 rpm as compare to higher speed for SiC, Al_2_O_3_, Cr and Si Powder. The order of hardness was found as follows: SiC > Al_2_O_3_ > Cr > Si. The FSPed area now has a hardness of 65 HRB, which is 50% higher than the base material, and is an improvement over it. This is due to higher speed enhance the temperature of internal section^54,55^. In a previous study^56^, it was already found that the alumina is a better reinforcement for the AA6101-T6 and AA1350 alloys.

**Figure 10:**
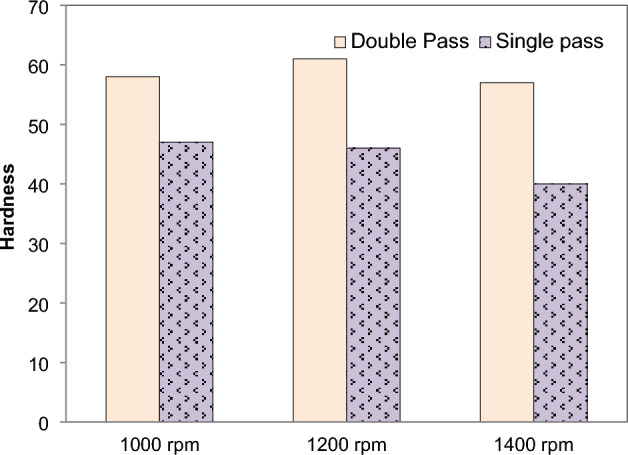
Effect of FSP passes on hardness (without reinforcement).

A larger value of hardness is being shown in the welded area as a result of the action of double pass, as shown in Fig. 10. The hardness of AZ31 FSPed composite was found ~ 4.4 to 3.05 times in double pass as compared to the single pass for the increase in rotational speed. There is no difference in the rating of hardness between single pass and double pass FSPed plates when it comes to chromium particle FSPed plates. When compared to the single pass FSPed area, the double pass with greater grain refinement yields superior results. This is something that we can claim with certainty. On the other hand, Si powder exhibits a lower value of hardness simply due to the fact that it has a lower level of hardness in comparison to other reinforced particles. Kumar et al.^57^ also revealed that the FSPed properties of the AA6101-T6 and AA1350 were achieved superior during the dual pass.

### Impact strength

**Figure 11:**
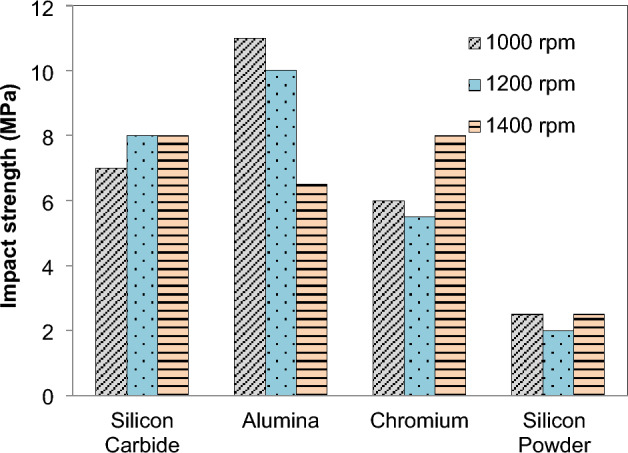
Effect of *N* on impact strength.

**Figure 12:**
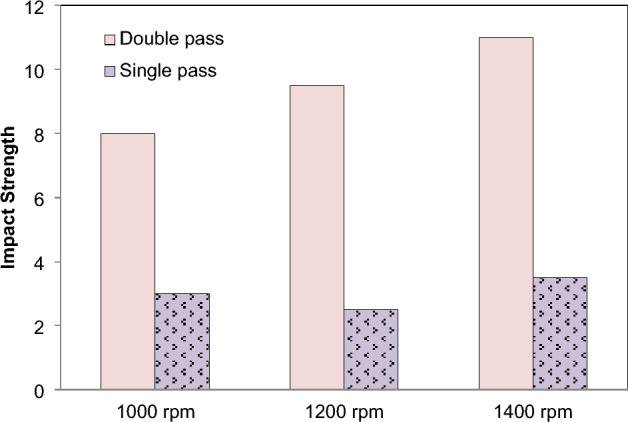
Effect of FSP passes on impact strength (without reinforcement).

Investigating the impact strength deviation of both the reinforced and base matrix FSPed specimens revealed that the material bends, deforms, and does not completely break down, indicating that it is delicate. At the stirred region zone, which is defined by the notch in the processed zone and the base plate, the impact strength of FSPed samples is measured. The impact strength varies material to material as the speed increases shown in Fig. 11. A base plate with impact strength of 2 J is inferior than the FSPed zone, which has an impact strength of 11.5 J. The impact strength of the Al_2_O_3_ FSPed area is much higher than that of silicon carbide particles. By incorporating various reinforcements into the matrix material, the composite's impact strength is enhanced. Without reinforcement, when single and double passes are made, the impact strength of the composite material is significantly affected, as shown in Fig. 12. The impact steength of AZ31 FSPed composite was increased by ~ 3.25 times in double pass as compared to the single pass for the increase in rotational speed. Because of the greater grain refinement achieved by the double passes FSPed plates in comparison to the single passes in the matrix metal, the impact strength of the double passes FSPed plates is superior to that of the single passes FSPed plates. To a considerable degree, the impact strength is not much affected by speed.

### Tensile strength results

**Figure 13:**
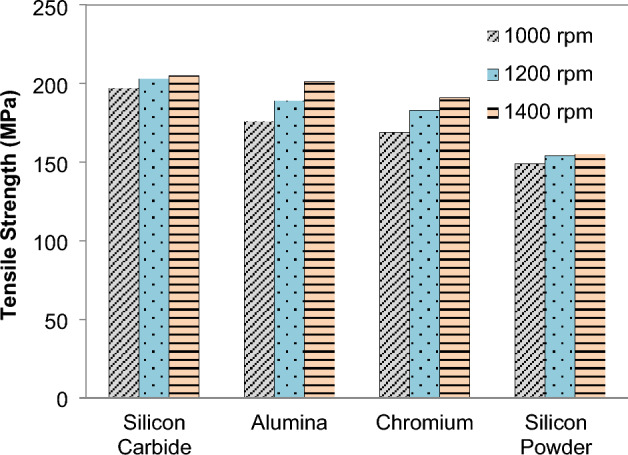
Effect of *N* on tensile strength.

**Figure 14:**
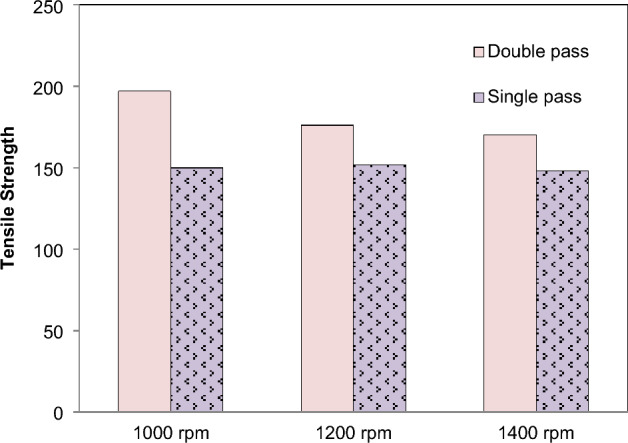
Effect of FSP passes on tensile strength (without reinforcement).

Wire electrical discharge machining (EDM) is used to cut the tensile samples, which determines the section size. To ensure that the tensile section gauge segment is in the middle of the three sections, the specifications are preferable. This is a standard mechanical test that measures the practical load by loading a carefully regulated set in a controlled environment^58^. The primary factors influencing the composite's tensile strength are the speed and the particle of reinforcements. For tensile strengths between 148 and 205 MPa, see Fig. 13. A tensile strength of 125 MPa was recorded for the base material. The increase in number of passes resulted in an increase in the composite's tensile strength. According to the results, the tensile strength of the two passes FSPed plates is much higher than that of the single section without any reinforcing, as revealed in previous study also^20^. When compared to speeds of 1200 and 1400 revolutions per minute, Fig. 14 demonstrate that double pass and single pass have a higher tensile strength at a speed of 1000 revolutions per minute. In litertaure, Alumina is found as a better reinforcement in terms of improving the tensile strength of the AA6101-T6, AA1350, and AA5052 alloys^59,60^.

### Microstructure of FSPed samples

**Figure 15:**
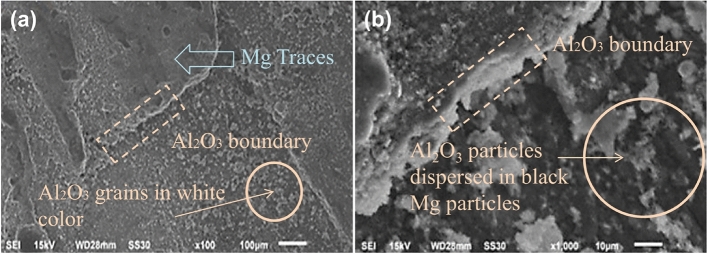
SEM photograph of Al_2_O_3_ reinforced composite at speed 1000 rpm in (**a**) × 100 magnification, (**b**) × 1000 magnification.

The microstructure of Al_2_O_3_ reinforced composite is shown in Fig. 15. The parameters are optimum as *N* = 1000 rpm and *r* = 40 mm/min. A very fine homogeneous microstructure of Al_2_O_3_ particles is observed at × 100 magnification, as shown in Fig. 15a. In Fig. 15b, as the magnification increases to × 1000 the mixing of Al_2_O_3_ particles with Mg in stirred region is observed. The white region shows the Al_2_O_3_ particles dispersed in black Mg particles. Because of higher melting point of Al_2_O_3_, Mg melts earlier in FSP process which is results in squeezed Al_2_O_3_ white particles between layers of Mg grains.

**Figure 16:**
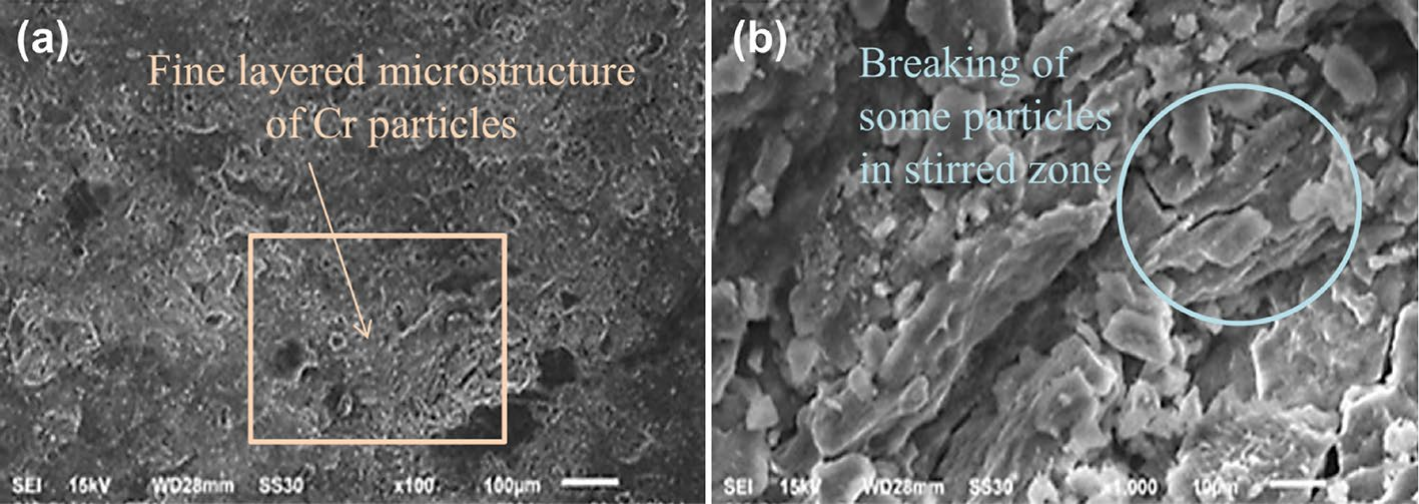
SEM photograph of Cr reinforced composite at speed 1000 rpm in (**a**) × 100 magnification and (**b**) × 1000 magnification.

In Fig. 16a, a fine but layered microstructure of Cr particles is observed at a × 100 magnification. At the magnification of × 1000 of Cr particles mixed with Mg particles shows large white–gray particles and black layers of Mg, from Fig. 16b. It observed that breaking of some particles in stirred zone and this is due to the stirring action generated in pass by the rotated probe in the matrix**.** The microstructure after dispersion is tends to more homogeneous. The fluctuation in heat produced during the stir processing affects the proportion of growth, dissolution, and reprecipitation of the precipitate^1,2,28,61,62^.

**Figure 17:**
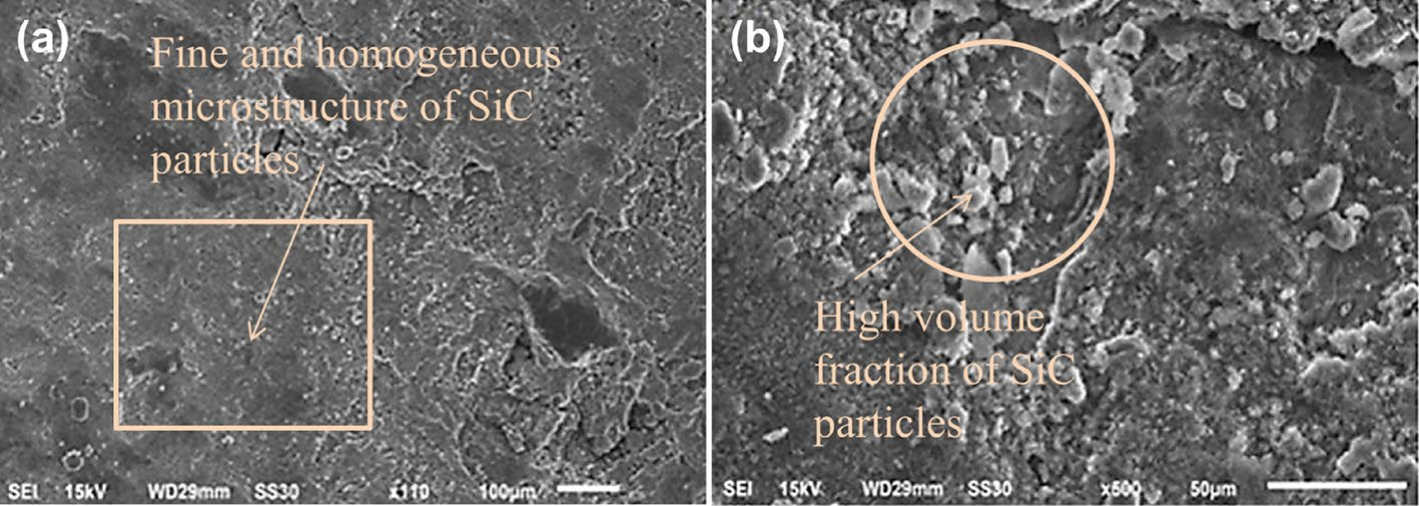
SEM photograph of SiC reinforced composite at speed 1000 rpm in (**a**) × 100 magnification and (**b**) × 500 magnification.

From Fig. 17a, a more fine and homogeneous microstructure of SiC particles is observed as compared to Cr, at a × 100 magnification. As the magnification increases to × 500, SiC particles seen as the white dots which represents the high volume fraction of SiC particles in contrast Mg particles, observed in Fig. 17b. SEM photographs are necessary to reveal the reinforcement particles that may appearance after the FSPed process. Since the reinforced particles are small and it is rigid to observe with optical microscopy. Similar was found by Li et al.^63^. Consequently, the FSP area has achieved finer grains with a uniform distribution, hence promoting the creation of FSPed joints without any defects^64,65^.

**Figure 18:**
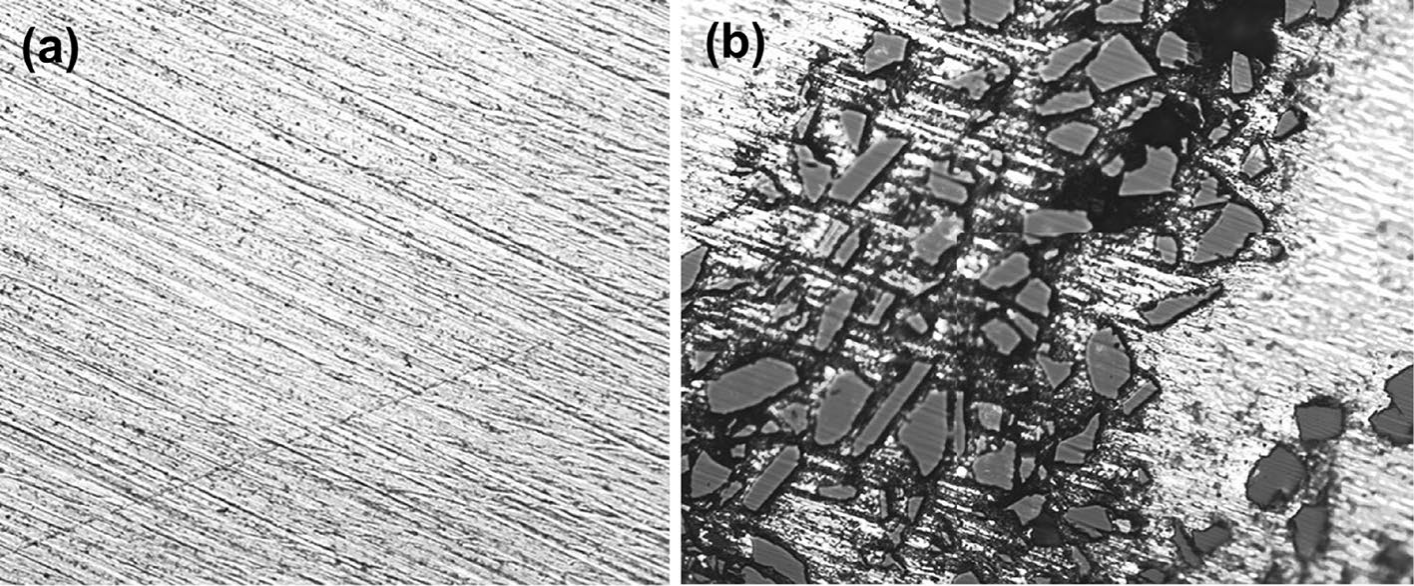
Microstructure images at × 500 magnification of (**a**) base matrix (AZ31) (**b**) after FSPed stir region with SiC reinforced composite at speed 1000 rpm.

Silicon carbide FSPed region at optimum speed of 1000 rpm have even distribution of reinforcement and this distribution can be seen in × 500 magnified optical microscopy photographs as shown in Fig. 18. Magnesium matrices have grains with different sizes. This is due to the effect of cooling during FSP process. The regions that appear grey in color are composed of new fine grains with Nano-SiC particles. It is evidently seen that refine grains are developed in stirred zone comparing with original grains in the matrix. It is possible that this indicates that the formation of these tiny and equiaxed grains occurs during the FSP process as a result of the action of dynamic recrystallization. Because no fractures nor exfoliations were found on the interface, it may be concluded that the fine-grained layers and the coarse-grained matrix are successfully bonded to one another. The agitated zone included SiC particles that were distributed in a rather regular manner.

**Figure 19:**
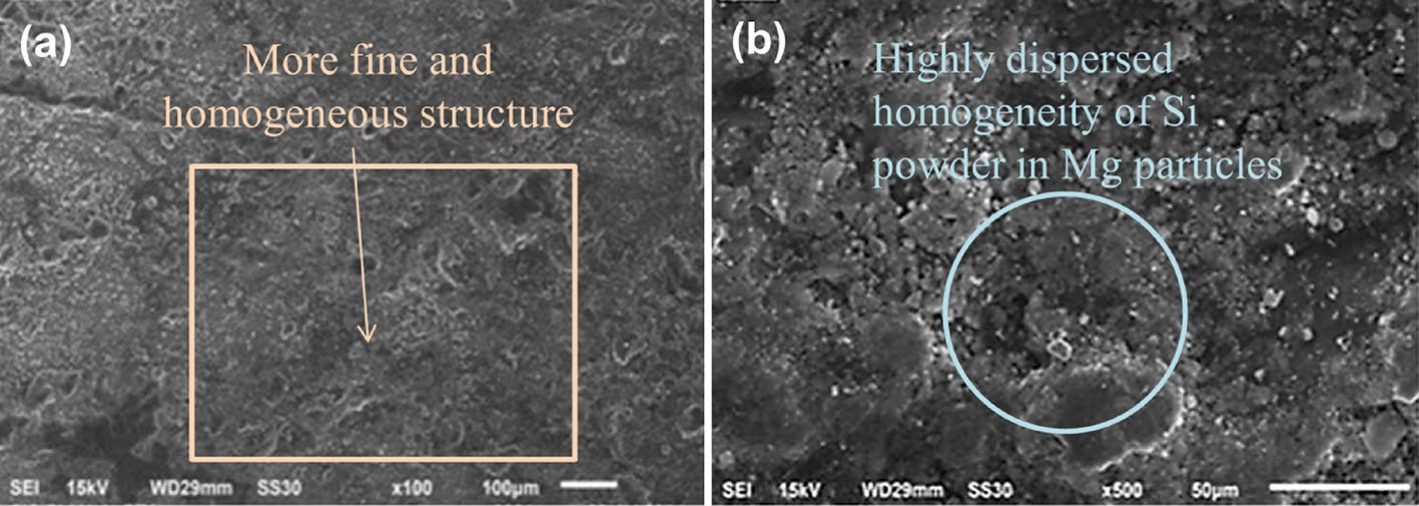
SEM photograph of Si Powder reinforced composite at speed 1000 rpm in (**a**) × 100 magnification and (**b**) × 500 magnification.

In Fig. 19a, Si powder dispersion in Mg particles shows much more fine structure as compare to Al_2_O_3_, SiC and Cr. At magnification of × 500, Fig. 19b shows the highly dispersed homogeneity of Si powder in Mg particles without any imperfection.

## Conclusions

FSP of Magnesium based composite is performed and effects on microstructural and mechanical properties is evaluated. The reinforcement particles SiC, Al_2_O_3_, Cr and Si powder were dispersed in AZ31 and composites were successfully produced. It is concluded that:

- Magnesium composites are successfully fabricated at various speeds 1000, 1200, 1400 rpm. From the macroscopic approach i.e. by naked eye, it is observed that as the rotation speed of tool increases the smoothness of surface increases.
- The peak values of mechanical strength were observed in FSPed region at tool rotation speed of 1000 rpm and constant transverse speed 40 mm/min.
- Addition of reinforcement results in better microstructural and mechanical properties as compared to the AZ31 magnesium alloy.
- The mean hardness of base plate of magnesium is found 32 HRB. The order of hardness was found as follows: SiC > Al_2_O_3_ > Cr > Si.
- Mechanical testing revealed an increase in hardness, impact strength and tensile strength of AZ31 alloy with the addition of reinforcements. The hardness of FSPed region is increased up to 50% of base material.
- The hardness of AZ31 FSPed composite was found ~ 4.4 to 3.05 times in double pass as compared to the single pass for the increase in rotational speed.
- The impact steength of AZ31 FSPed composite was increased by ~ 3.25 times in double pass as compared to the single pass for the increase in rotational speed.
- The microstructure shows that reinforced particles were uniform dispersed into FSPed region and agglomerated with Mg matrix.
- Si powder produces finer microstructure as compare to SiC, Al_2_O_3_, Cr. FSP decreases the grain size of processed material.
- The tensile strength of the two passes FSPed plates is much higher than that of the single section without any reinforcement.

## Data Availability

The data is available within the manuscript.

## References

[1] Wu Y (2024). In-situ EBSD study on twinning activity caused by deep cryogenic treatment (DCT) for an as-cast AZ31 Mg alloy. J. Mater. Res. Technol..

[2] Li M (2022). Microstructure and properties of graphene nanoplatelets reinforced AZ91D matrix composites prepared by electromagnetic stirring casting. J. Mater. Res. Technol..

[3] Kumar D, Phanden RK, Thakur L (2021). A review on environment friendly and lightweight magnesium-based metal matrix composites and alloys. Mater. Today Proc..

[4] Friedrich HE, Mordike BL (2006). Magnesium Technology.

[5] Upadhyay G (2022). Development of carbon nanotube (CNT)-reinforced Mg alloys: Fabrication routes and mechanical properties. Metals.

[6] Sharma SK (2022). Significance of alloying elements on the mechanical characteristics of Mg-based materials for biomedical applications. Crystals.

[7] Prabhakar DAP (2022). A comprehensive review of friction stir techniques in structural materials and alloys: Challenges and trends. J. Mater. Res. Technol..

[8] Arora GS, Saxena KK, Mohammed KA, Prakash C, Dixit S (2022). Manufacturing techniques for Mg-based metal matrix composite with different reinforcements. Crystals.

[9] Deng J, Wu R, Sun Z, Qian D, Zhang Y (2024). A prediction model of ultimate forming dimension for profile ring with outer groove in ring rolling process. Int. J. Adv. Manuf. Technol..

[10] Li J (2024). Task incremental learning-driven digital-twin predictive modeling for customized metal forming product manufacturing process. Robot. Comput. Integr. Manuf..

[11] Su Y (2023). Gaussian filtering method of evaluating the elastic/elasto-plastic properties of sintered nanocomposites with quasi-continuous volume distribution. Mater. Sci. Eng. A.

[12] Long X (2023). An insight into dynamic properties of SAC305 lead-free solder under high strain rates and high temperatures. Int. J. Impact Eng..

[13] Zhang Z (2022). Effects of solder thickness on interface behavior and nanoindentation characteristics in Cu/Sn/Cu microbumps. Weld. World.

[14] Reddy KRRM, Ramanaiah N, Sarcar MMM (2016). Corrosion behavior of duplex coatings. J. King Saud. Univ. Eng. Sci..

[15] Song J (2024). Microstructure and mechanical properties of novel Ni–Cr–Co-based superalloy GTAW joints. J. Mater. Res. Technol..

[16] Wang J (2020). Evolution of crystallographic orientation, precipitation, phase transformation and mechanical properties realized by enhancing deposition current for dual-wire arc additive manufactured Ni-rich NiTi alloy. Addit. Manuf..

[17] Jiang XJ (2023). Effect of Zr on microstructure and properties of TC4 alloy fabricated by laser additive manufacturing. J. Mater. Res. Technol..

[18] Sharma V, Prakash U, Kumar BVM (2015). Surface composites by friction stir processing: A review. J. Mater. Process. Technol..

[19] Gong Z (2024). Effect of laser shock peening on stress corrosion cracking of TC4/2A14 dissimilar metal friction stir welding joints. J. Mater. Res. Technol..

[20] Fang JX (2019). Effects of phase transition temperature and preheating on residual stress in multi-pass & multi-layer laser metal deposition. J. Alloys Compd..

[21] Zhang D (2023). Electromagnetic shocking induced fatigue improvement via tailoring the α-grain boundary in metastable β titanium alloy bolts. J. Alloys Compd..

[22] Zhao Y, Jing J, Chen L, Xu F, Hou H (2021). Current research status of interface of ceramic-metal laminated composite material for armor protection. Acta Met. Sin..

[23] Shao L (2023). Effect of cold-spray parameters on surface roughness, thickness and adhesion of copper-based composite coating on aluminum alloy 6061 T6 substrate. Processes.

[24] Qian W (2024). In situ X-ray imaging and numerical modeling of damage accumulation in C/SiC composites at temperatures up to 1200 °C. J. Mater. Sci. Technol..

[25] Christener, B. K. & Sylva, G. D. Friction Stir weld developments for aerospace application’. in *International Conference on Advances in Welding Technology Joining of High Performance Materials*, 6–8 (1996).

[26] Darras, B. M. Experimental and analytical study of friction stir processing. (2005).

[27] Fang JX (2021). Transformation-induced strain of a low transformation temperature alloy with high hardness during laser metal deposition. J. Manuf. Process..

[28] Fang JX (2022). Microstructure evolution and deformation behavior during stretching of a compositionally inhomogeneous TWIP-TRIP cantor-like alloy by laser powder deposition. Mater. Sci. Eng. A.

[29] Wu Y (2022). Effect of boron on the structural stability, mechanical properties, and electronic structures of γ′-Ni3Al in TLP joints of nickel-based single-crystal alloys. Mater. Today Commun..

[30] Xie B, Li H, Ning Y, Fu M (2023). Discontinuous dynamic recrystallization and nucleation mechanisms associated with 2-, 3-and 4-grain junctions of polycrystalline nickel-based superalloys. Mater. Des..

[31] Zhao Y (2023). Understanding and design of metallic alloys guided by phase-field simulations. NPJ Comput. Mater..

[32] Morisada Y, Fujii H, Nagaoka T, Fukusumi M (2006). MWCNTs/AZ31 surface composites fabricated by friction stir processing. Mater. Sci. Eng. A.

[33] Chang CI, Lee CJ, Chuang CH, Pei HR, Huang JC (2007). On Mg–Al–Zn intermetallic alloys made by friction stir processing containing quasi-crystals or amorphous phases. Adv. Mater. Res..

[34] Dwivedi SP (2022). Alumina catalyst waste utilization for aluminum-based composites using the friction stir process. Mater. Test..

[35] Yc C, Nakata K (2009). Friction stir lap welding of magnesium alloy and zinc-coated steel. Mater. Trans..

[36] Kurt A, Uygur I, Cete E (2011). Surface modification of aluminium by friction stir processing. J. Mater. Process. Technol..

[37] Antony M, Pavithran BT, Thamban I (2013). Friction stir processing of AA6061: A study. Int. J. Emerg. Technol. Adv. Eng..

[38] Wais AMH, Salman JM, Al-Roubaiy AO (2013). Effect of friction stir processing on mechanical properties and microstructure of the cast pure aluminum. Int. J. Sci. Technol. Res..

[39] Kumar S, Kumar K, Maurya M (2021). Parametric optimization of friction stir processing on micro-hardness of Al/B4C composite. Int. J. Mater. Res..

[40] Devaraju A, Kumar A, Kumaraswamy A, Kotiveerachari B (2013). Influence of reinforcements (SiC and Al_2_O_3_) and rotational speed on wear and mechanical properties of aluminum alloy 6061–T6 based surface hybrid composites produced via friction stir processing. Mater. Des..

[41] Aonuma M, Nakata K (2011). Dissimilar metal joining of 2024 and 7075 aluminium alloys to titanium alloys by friction stir welding. Mater. Trans..

[42] Sinhmar S, Dwivedi DK, Pancholi V (2014). Friction stir processing of AA 7039 alloy. Int. Conf. Prod. Mech. Eng..

[43] Huang Y, Wang T, Guo W, Wan L, Lv S (2014). Microstructure and surface mechanical property of AZ31 Mg/SiCp surface composite fabricated by direct friction stir processing. Mater. Des..

[44] Kumar RA, Thansekhar MR (2018). Reinforcement with alumina particles at the interface region of AA6101-T6 and AA1350 alloys during friction stir welding. Mater. Res. Express.

[45] Kumar RA (2019). Effect of hybrid reinforcement at stirred zone of dissimilar aluminium alloys during friction stir welding. Metall. Res. Technol..

[46] Dwivedi SP, Maurya NK, Maurya M, Saxena A, Srivastava AK (2021). Optimization of casting parameters for improved mechanical properties of eggshell reinforced composites. Mater. Test..

[47] Hua L (2022). Mechanism of void healing in cold rolled aeroengine M50 bearing steel under electroshocking treatment: A combined experimental and simulation study. Mater. Charact..

[48] Ji R (2023). Study on high wear resistance surface texture of electrical discharge machining based on a new water-in-oil working fluid. Tribol. Int..

[49] Gao S, Li H, Huang H, Kang R (2022). Grinding and lapping induced surface integrity of silicon wafers and its effect on chemical mechanical polishing. Appl. Surf. Sci..

[50] Tang D (2024). On the nonlinear time-varying mixed lubrication for coupled spiral microgroove water-lubricated bearings with mass conservation cavitation. Tribol. Int..

[51] Gong, Q. *et al.* Grinding surface and subsurface stress load of nickel-based single crystal superalloy DD5. *Precis. Eng.* (2024).

[52] Gupta MK (2020). Friction stir process: A green fabrication technique for surface composites: A review paper. SN Appl. Sci..

[53] Almazrouee AI, Al-Fadhalah KJ, Alhajeri SN (2021). A new approach to direct friction stir processing for fabricating surface composites. Crystals.

[54] Maurya M, Kumar S, Bajpai V (2018). Assessment of the mechanical properties of aluminium metal matrix composite: A review. J. Reinf. Plast. Compos..

[55] Santhosh MS, Natrayan L, Kaliappan S (2022). Mechanical and wear behavior of nano fly-ash particle-reinforced mg metal matrix composites fabricated by stir cast technique. J. Nanomater..

[56] Kumar AR (2023). Role of traversing speed and axial load on the properties of friction stir welded dissimilar AA6101-T6 AND AA1350 aluminium alloys. J. Chin. Inst. Eng..

[57] Ashok Kumar R, Thansekhar MR (2018). Mechanical and wear properties of friction stir welded dissimilar AA6101-T6 and AA1350 alloys: Effect of offset distance and number of passes. J. Mech. Sci. Technol..

[58] Surakasi R, Paramasivam P, Dhanasekaran S, Patil PP (2023). Statistical experiment analysis of wear and mechanical behaviour of abaca/sisal fiber-based hybrid composites under liquid nitrogen environment. Front. Mater..

[59] Kasirajan G, Rengarajan S, Raghav GR, Rao VS, Nagarajan KJ (2020). Tensile and wear behaviour of friction stir welded AA5052 and AA6101-T6 aluminium alloys: Effect of welding parameters. Metall. Res. Technol..

[60] Muneeswaran R, Mohan MS, Rengarajan S, Raghav GR, Nagarajan KJ (2020). Effects of tool pin profile on tensile and wear behaviour of friction stir welded AA6101-T6 and AA1350 alloys. Metall. Res. Technol..

[61] Kumar RA, Thansekhar MR (2019). Wear behaviour of friction stir welded dissimilar aluminium alloys. Met. Noveishie Tekhnol..

[62] Gao Q, Ding Z, Liao W-H (2022). Effective elastic properties of irregular auxetic structures. Compos. Struct..

[63] Li H (2024). Abrasion performance and failure mechanism of fiber yarns based on molecular segmental differences. J. Eng. Fiber. Fabr..

[64] Kumar RA, Thansekhar MR (2017). Property evaluation of friction stir welded dissimilar metals: AA6101-T6 and AA1350 aluminium alloys. Mater. Sci..

[65] Zhu Q, Chen J, Gou G, Chen H, Li P (2017). Ameliorated longitudinal critically refracted: Attenuation velocity method for welding residual stress measurement. J. Mater. Process. Technol..

